# Progress and Perspective of Electrocatalytic CO_2_ Reduction for Renewable Carbonaceous Fuels and Chemicals

**DOI:** 10.1002/advs.201700275

**Published:** 2017-09-29

**Authors:** Wenjun Zhang, Yi Hu, Lianbo Ma, Guoyin Zhu, Yanrong Wang, Xiaolan Xue, Renpeng Chen, Songyuan Yang, Zhong Jin

**Affiliations:** ^1^ Key Laboratory of Mesoscopic Chemistry of MOE School of Chemistry and Chemical Engineering Nanjing University Nanjing 210023 China

**Keywords:** carbon cycle, catalytic mechanisms, electrochemical CO_2_ reduction, electrocatalysts, renewable fuels

## Abstract

The worldwide unrestrained emission of carbon dioxide (CO_2_) has caused serious environmental pollution and climate change issues. For the sustainable development of human civilization, it is very desirable to convert CO_2_ to renewable fuels through clean and economical chemical processes. Recently, electrocatalytic CO_2_ conversion is regarded as a prospective pathway for the recycling of carbon resource and the generation of sustainable fuels. In this review, recent research advances in electrocatalytic CO_2_ reduction are summarized from both experimental and theoretical aspects. The referred electrocatalysts are divided into different classes, including metal–organic complexes, metals, metal alloys, inorganic metal compounds and carbon‐based metal‐free nanomaterials. Moreover, the selective formation processes of different reductive products, such as formic acid/formate (HCOOH/HCOO^−^), monoxide carbon (CO), formaldehyde (HCHO), methane (CH_4_), ethylene (C_2_H_4_), methanol (CH_3_OH), ethanol (CH_3_CH_2_OH), etc. are introduced in detail, respectively. Owing to the limited energy efficiency, unmanageable selectivity, low stability, and indeterminate mechanisms of electrocatalytic CO_2_ reduction, there are still many tough challenges need to be addressed. In view of this, the current research trends to overcome these obstacles in CO_2_ electroreduction field are summarized. We expect that this review will provide new insights into the further technique development and practical applications of CO_2_ electroreduction.

## 1 Introduction

The urgent energy crisis and serious global warming problem represent two major challenges of the world. In the past decades, tremendous efforts have been made to relieve these issues. Most energy consumed by human society was derived from nonrenewable fossil fuels.^1^, ^2^ Carbon dioxide (CO_2_) is an extremely disturbing greenhouse gas released from the excessive use of fossil fuels. The CO_2_ emission problems have drawn intensive attention and increasing investments for more than 30 years. CO_2_ gas produced on the Earth should be equal to the amount consumed, so that the concentration of CO_2_ in atmosphere can remain unchanged to realize eco‐environmental stability and a favorable transition toward a sustainable society. Decreasing CO_2_ emissions and further regenerating CO_2_ into carbonaceous fuels and chemicals by mimicking the photosynthesis process of green plants would be an excellent method to relieve our demands on high‐polluting fossil energy and provide indispensable resources for industrial applications.^3^, ^4^, ^5^

Compared to the geological sequestration of CO_2_, to convert waste CO_2_ gas into hydrocarbons is recognized as a more worthwhile approach owing to its high‐efficiency utilization and recycling of carbon sources. To achieve this goal, the traditional catalytic processes of CO_2_ absorption, activation, and conversion still suffer from certain drawbacks, such as high energy demands for the transfer of CO_2_ molecules to active sites, low conversion rate to obtain high‐value carbonaceous chemicals, and so on. So far, various methods have been adopted to convert CO_2_ into other chemicals, such as: (1) biologic transformation with microalgae outdoor‐pool/photobioreactor or biocatalysis;^6^, ^7^, ^8^, ^9^ (2) chemical transformation through organic reactions or mineralization/carbonatation;^10^, ^11^, ^12^ (3) photocatalytic or electrocatalytic reduction;^13^, ^14^, ^15^, ^16^ and (4) other techniques like hydrogenation, dry reforming, and so on.^17^, ^18^, ^19^, ^20^ It is worthy mentioned that the realization of CO_2_ reduction by electrochemical catalysis has attracted great attention owing to the unique merits,^21^, ^22^, ^23^, ^24^, ^25^, ^26^ as follows: (1) the CO_2_ electroreduction system can be employed for practical application; (2) the electrocatalytic process under mild conditions is moderate and controllable; (3) the products of electrochemical reduction can be adjusted by reaction parameters, such as redox potential, reaction temperature, electrolyte, etc.; (4) through the optimization of electrocatalysts, the by‐products of CO_2_ reduction can be minimized to a low content; (5) electric power, as the drive force, can be attained with other renewable energy sources (such as solar power, wind power, and so on) without any additional CO_2_ generation.

**Scheme 1:**
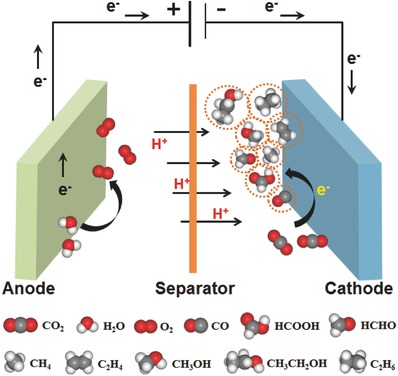
Illustration of the electrochemical CO_2_ reduction process and the possible products generated in an electrochemical reaction cell.

CO_2_ molecules are very inert and stable, because the carbon atoms in CO_2_ are at the highest oxidation state. Therefore, it is necessary to develop efficient electrocatalysts for promoting the kinetically sluggish CO_2_ reduction process. The routes of electrochemical CO_2_ reduction can be realized through multiple electron transfer in aqueous solution with suitable electrocatalysts. The various possible products formed through different pathways in a schematic electrocatalytic cell are presented in **Scheme** **1**. CO_2_ can be converted into small carbonaceous molecules with high energy density, such as formic acid (HCOOH), carbon monoxide (CO), methanol (CH_3_OH), methane (CH_4_), and so on. Based on a thermodynamic study, a variety of half‐reactions and their corresponding electrode potentials versus standard hydrogen electrode (SHE) in aqueous solution (pH = 7, at 25 °C, 1 atm, and 1.0 m concentration of other solutes) are listed in **Table** **1**.^15^, ^27^ It is very likely that a mixture composed of gaseous products (CO, CH_4_, etc.) and liquid products (HCOOH, CH_3_OH, C_2_H_5_OH, etc.) would be formed in the electrochemical cell, rather than a single product. The employed electrocatalysts and the applied electrode potential are crucial to the efficiency and selectivity of CO_2_ reduction. This brings some serious pending technological challenges, such as high cost, inferior efficiency, low product selectivity, and fast degradation of electrocatalytic activity.^28^, ^29^, ^30^ Especially, due to the inadequate selectivity and stability of existing electrocatalysts, the state‐of‐the‐art techniques are still unable to adequately meet the requirements for large‐scale industrial application.

**Table 1:** Electrochemical potentials of possible CO_2_ reduction reactions in aqueous solutions for the production of different hydrocarbon fuels

Possible half‐reactions of electrochemical CO_2_ reduction	Electrode potentials (V vs SHE) at pH 7
CO_2_ (g) + e^−^ → *COO^−^	−1.90
CO_2_ (g) + 2H^+^ + 2e^−^ → HCOOH (l)	−0.61
CO_2_ (g) + H_2_O (l) + 2e^−^ → HCOO^−^ (aq) + OH^−^	−0.43
CO_2_ (g) + 2H^+^ + 2e^−^ → CO (g) + H_2_O (l)	−0.53
CO_2_ (g) + H_2_O (l) + 2e^−^ → CO (g) + 2OH^−^	−0.52
CO_2_ (g) + 4H^+^ + 2e^−^ → HCHO (l) + H_2_O (l)	−0.48
CO_2_ (g) + 3H_2_O (l) + 4e^−^ → HCHO (l) + 4OH^−^	−0.89
CO_2_ (g) + 6H^+^ (l) + 6e^−^ → CH_3_OH (l) + H_2_O (l)	−0.38
CO_2_ (g) + 5H_2_O (l) + 6e^−^ → CH_3_OH (l) + 6OH^−^	−0.81
CO_2_ (g) + 8H^+^ + 8e^−^ → CH_4_ (g) + 2H_2_O (l)	−0.24
CO_2_ (g) + 6H_2_O (l) + 8e^−^ → CH_4_ (g) + 8OH^−^	−0.25
2CO_2_ (g) + 12H^+^ + 12e^−^ → C_2_H_4_ (g) + 4H_2_O (l)	0.06
2CO_2_ (g) + 8H_2_O (l) + 12e^−^ → C_2_H_4_ (g) + 12OH^−^	−0.34
2CO_2_ (g) + 12H^+^ + 12e^−^ → CH_3_CH_2_OH (l) + 3H_2_O (l)	0.08
2CO_2_ (g) + 9H_2_O (l) + 12e^−^ → CH_3_CH_2_OH (l) + 12OH^−^ (l)	−0.33

In the past decades, numerous efforts have been made to ameliorate the electrocatalysts and reaction conditions to overcome the above obstacles.^15^, ^16^, ^26^, ^31^, ^32^, ^33^ In recent years, as the demand of clean energy is increasing worldwide, the research of CO_2_ electroreduction is progressing very rapidly. Therefore, a comprehensive review includes various aspects, such as electrocatalysts categories, product selectivity, stability, as well as challenges and perspectives, is needed for summarizing the recent advances and promoting the further development in this field.

## 2 Electrocatalysts for Electrocatalytic CO_2_ Reduction

The electrocatalysts applicable to CO_2_ reduction can be classified into different types, basically inorganic and organic species. Since the 1970s, some metal–organic complexes have been applied as a class of typical homogeneous electrocatalysts, because their special coordinative structures and active centers can tightly bind with CO_2_ molecules.^34^ The electrocatalysts based on metal–organic complexes have attracted significant attention for decades due to the remarkable selectivity, but also have some unpopular disadvantages, such as complicate synthesis processes, low reduction activity, and toxic effects.^35^, ^36^ Heterogeneous metal electrocatalysts have been developed later, accompanying with some advantageous characteristics, such as low toxicity, facile synthesis processes and superior electrocatalytic activity.^37^, ^38^ Inorganic metal compounds (metal oxides, chalcogenides, etc.) and carbon‐based materials have also been employed as emergent electrocatalysts. The following sections will introduce the development of these representative electrocatalysts of CO_2_ reduction in recent five years.

### 2.1 Metal–Organic Complexes

#### 2.1.1 Metal–Macrocyclic Complexes

**Figure 1:**
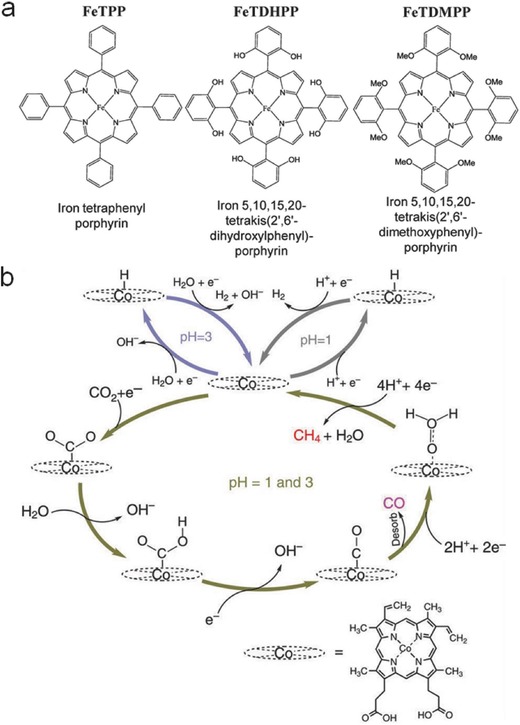
Metal–macrocyclic complexes as electrocatalysts for CO_2_ reduction. a) Investigated iron porphyrins. Reproduced with permission.^39^ Copyright 2016, American Association for the Advancement of Science. b) Schematic mechanism of the electrochemical CO_2_ reduction using Co protoporphyrin. Reproduced with permission.^40^ Copyright 2015, Macmillan Publishers Limited.

Macrocyclic ligands can be divided into different classes, such as phthalocyanine, porphyrin, cyclam, and so on. In 1970s, Meshitsuka et al. first reported the utilization of metal–macrocyclic complexes composed of transition metal atom (Co or Ni) and phthalocyanine ligands for CO_2_ electroreduction.^34^ Since then, numerous of researches related to metal–macrocyclic complexes have been come forth. Acted as an applicable and desirable “Trash to Treasure” approach,^39^ the greenhouse gas CO_2_ can be effectively transferred into carbon monoxide (CO) using different kinds of Fe‐porphyrin molecules, as illustrated in **Figure** **1**a. Typically, iron 5,10,15,20‐tetrakis(2′,6′‐dihydroxylphenyl)‐porphyrin (Fe TDHPP) could achieve a stable electrocatalytic performance over 4 h for CO generation with a Faradaic yield above 90%, attributing to the high local proton concentration of phenolic hydroxyl. A cobalt‐protoporphyrin electrocatalyst loaded on pyrolytic graphite electrode can convert CO_2_ mainly into CO in acidic conditions,^40^ showing high electrocatalytic activity comparable to other porphyrin‐based molecules in previous reports at a lower overpotential (0.5 V). The pH‐dependent activity and selectivity are shown in Figure 1b. Besides, a composite electrode prepared by the electrodeposition of [Cu(cyclam)](ClO_4_)_2_ complex (cyclam = 1,4,8,11‐tetraazacyclotetradecane) can reduce CO_2_ into HCOOH with a Faradaic efficiency of 90% in dimethyl formamide (DMF)/H_2_O mixture (97:3, v/v).^41^

#### 2.1.2 Metal–Bipyridine Complexes

**Figure 2:**
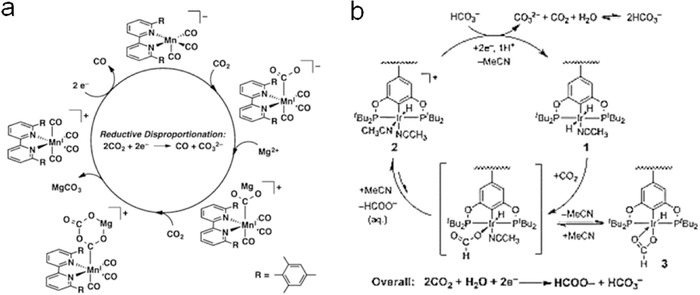
a) Redox mechanism of [Mn(mesbpy)(CO)_3_]^−^ and Mg^2+^ at −1.5 V versus Fc^+/0^ for electroreduction of CO_2_ to CO. Reproduced with permission.^52^ Copyright 2016, American Chemical Society. b) Proposed mechanism for electroreduction of CO_2_ to HCOO^−^ using iridium pincer dihydride electrocatalyst. Reproduced with permission.^54^

Bipyridine (bpy) complexes with earth‐abundant metal atoms were also considered as promising molecular electrocatalysts for reducing CO_2_ to CO or hydrocarbons, such as HCOOH. There are many transition metals explored in this group, such as Ru, Cu, W, Mo, Mn, Re, Cr, and so on.^42^, ^43^, ^44^, ^45^, ^46^, ^47^, ^48^, ^49^, ^50^, ^51^, ^52^ For instance, a metal complex composed of Ru atom and 6,6′‐dimesityl‐2,2′‐bipyridine (mesbpy) ligands was applied to the generation of CO with high turnover frequency and Faradaic efficiency in the presence of Brønsted acids.^51^ The results benefited from the inhibition of Ru—Ru bond formation as well as the synergistic redox response between bipyridine ligands and Ru metal. Similarly, a manganese (Mn) based complex electrocatalyst composed of mesbpy ligands shows good performance at low overpotentials (0.3–0.45 V) with the assistance of Lewis acid (especially Mg^2+^ cations).^52^ The electrocatalytic mechanism of [Mn(mesbpy)(CO)_3_]^−^ for converting CO_2_ into CO was detailed presented in **Figure** **2**a.

#### 2.1.3 Other Metal–Organic Complexes

Some other organic–ligand based complexes have also been investigated as molecular electrocatalysts. Donovan et al. synthesized two new Zn(II) complexes with phosphine groups and evaluated their ability to reduce CO_2_ to CO.^53^ Kang et al. reported an iridium pincer dihydride electrocatalysts adopted to reduce CO_2_ to formates (HCOO^−^),^54^ exhibiting high efficiency, selectivity and turnover numbers (≈54200), of which mechanism is shown in Figure 2b. Besides, molecular electrocatalysts with other components, such as biscarbene pincer,^55^ *N*‐heterocyclic carbene,^56^ polyaniline,^57^ (R,R)‐Trost‐bis‐ProPhenol ([BPP]),^58^ 4‐v‐tpy, 6‐v‐tpy,^59^ oxalate,^60^ and hydride,^61^ were also studied. Inspired by these analogous researches, the future extensive exploration of metal complex catalysts is to be expected.

### 2.2 Metals

**Figure 3:**
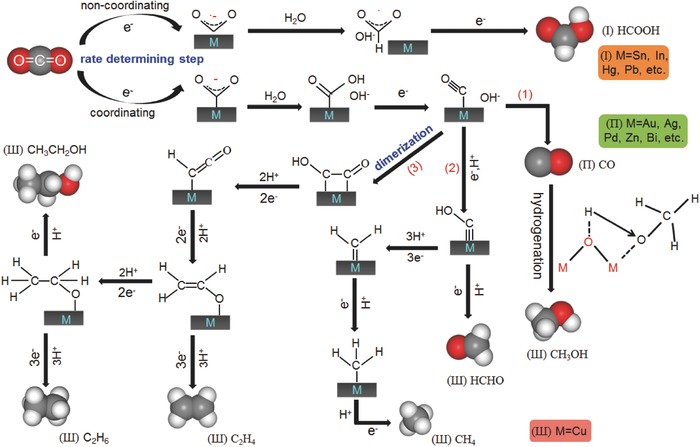
Schematic mechanism of different metal electrocatalysts for CO_2_ reduction reaction in aqueous solution.

Metal electrocatalysts for CO_2_ reduction can be divided into three groups based on the different reaction routes and main products (CO, HCOO^−^, hydrocarbons, alcohols, and so on), as illustrated in **Figure** **3**. Sn and Pb metals are classified as the same class, because they mainly generate HCOO^−^ in aqueous solution since CO_2_•^−^ intermediates can be easily desorbed from the surface of Sn and Pb.^62^ In comparison, Au, Ag, Pd, Zn, and Bi can tightly bind with *COOH intermediates, but can hardly bind with the generated *CO species, hence this class of metals tends to generate CO as the predominant product.^63^ Specially, Cu metal is individually divided into the third class, because Cu is in favor of binding *CO intermediates and converting it into alcohols or other hydrocarbons from *COH or *CHO intermediates through dimerization pathways.^64^ It is also worthy mentioned that some other metals like Pt, Ni have lower hydrogen evolution overpotentials and strong binding capability with *CO intermediates,^65^, ^66^ therefore the H_2_ evolution reaction (HER) will be the predominant process in the presence of water. Based on the chief principle of catalytic process concerning metallic catalysts, the concept of electronic structure should be introduced to pursue more anticipative performance.^67^ The key factor underlying the catalytic mechanism is that the interaction between adsorbate (CO_2_ molecules in this case) and metal surface are enormously determined by the d‐band levels of the catalyst itself. By adjusting the location of the d band centers, the bonding strength of adsorbed intermediates (*COOH, *CO, etc.) and Gibbs free energy (ΔG) consumed in rate‐determining steps would be optimized to enhance the catalytic performance. Hence, achieving satisfying activities of metallic catalysts relies on the adjustment of d band levels through lots of approaches,^68^, ^69^ such as particle size optimization, surface modification, and exposure of different crystal planes/active sites (such as terraces, edges or corners), etc. The recent progresses on different classes of metal catalysts for CO_2_ electroreduction are introduced in detail below.

#### 2.2.1 Sn and Pb

Sn and Pb, as group IV metals, are categorized as the first class of metal catalysts. In most cases, Sn and Pb mainly produce HCOO^−^ or formic acid owing to their weak bonding with CO_2_•^−^ intermediates.^62^ In recent years, there are some researches in the electrochemical performance of Sn and Pb electrodes with different parameters, such as electrocatalyst sizes, surface modification and reaction conditions. In regard of the effect of particulate sizes, Castillo et al. showed that smaller Sn nanoparticles (NPs) were helpful to overcome the mass transfer limitation of CO_2_ onto the electrode surface and reached an enhanced Faradaic efficiency for HCOO^−^ generation.^70^ Some surface modifications on Sn electrodes were also carried out to improve the activity and selectivity. It was reported that rationally designed SnO*_x_*‐derived Sn electrodes could electroreduce CO_2_ into HCOOH with superior Faradaic efficiencies and high production rates at relatively low overpotentials.^71^, ^72^, ^73^, ^74^, ^75^ The results indicated that the CO_2_•^−^ intermediates were preferably stabilized on the surface of Sn with abundant oxygen species rather than on bare Sn electrodes. Wu et al. investigated the obvious difference of HCOOH generation rates using Sn electrode in Na_2_SO_4_ and KHCO_3_ electrolytes, respectively, emphasizing the nonnegligible effect of reaction conditions.^76^

Recently, Zhu et al. fabricated Pb electrodes for CO_2_ electroreduction,^77^ showing high partial current density and Faradaic efficiency of HCOOH production in an ionic liquid/acetonitrile/H_2_O ternary electrolyte. The above‐mentioned researches about earth‐abundant electrodes like Sn and Pb may provide a feasible pathway for the noteworthy yield of HCOOH through the optimization of metal catalysts and electrolytes.

#### 2.2.2 Au, Ag, Pd, Zn, and Bi

**Figure 4:**
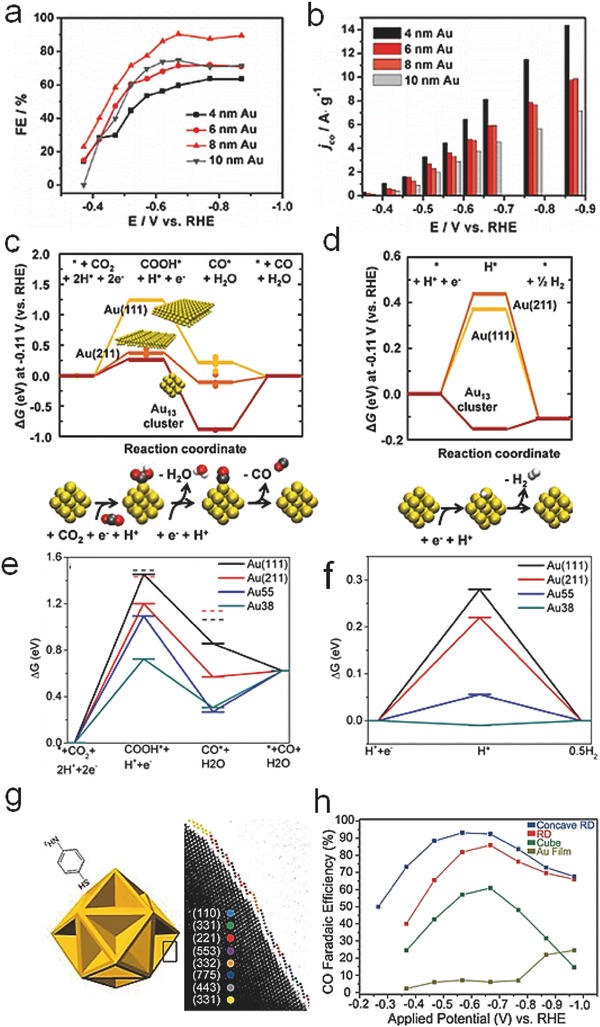
a) Potential‐dependent Faradaic efficiencies of different Au NPs (4, 6, 8, 10 nm) during electrocatalytic reduction of CO_2_ to CO. b) Current densities (mass activity) for electrocatalytic reduction of CO_2_ to CO on the Au NPs with different sizes at various applied potentials. Free energy diagrams for electrochemical reduction of c) CO_2_ to CO and d) protons to hydrogen on Au (111), Au (211), and a 13‐atom Au cluster at −0.11 V (vs RHE), respectively. Reproduced with permission.^79^ Copyright 2013, American Chemical Society. Free energy diagrams for electrochemical reduction of e) CO_2_ to CO and f) H^+^ to H_2_ on Au(111), Au(211), Au_55_ NPs, and Au_38_ NPs at 0 V versus RHE. Reproduced with permission.^80^ Copyright 2014, American Chemical Society. g) Morphological model of concave rhombic dodecahedron Au NPs with different exposed facets. h) Faradaic efficiencies of different Au NPs and Au film for CO production at applied potential (vs RHE). Reproduced with permission.^81^ Copyright 2015, American Chemical Society.

The second class of metals, such as Au, Ag, Pd, Zn, and Bi has aroused intense attention for the specific selectivity of CO generation. The following works have verified that ligand‐protected Au clusters and NPs with various sizes, exposed planes or special morphologies can exhibit distinctive catalytic activity and selectivity.^78^, ^79^, ^80^, ^81^ Kauffman et al. reported Au_25_ clusters could effectively realize the reduction of CO_2_ into CO with ≈100% Faradaic efficiency, indicating a reversible interaction between CO_2_ and Au_25_.^78^ Monodisperse Au NPs with size‐dependent electrocatalytic activity were also synthesized to achieve superior Faradaic efficiency for selective CO production (**Figure** **4**a–d).^79^ The extraordinary selectivity of CO strongly depends on the binding energies of different reaction intermediates on active sites. The competitive processes of CO and H_2_ generation are displayed as following

(1) $${\mathrm{CO}}_{2}\left(g\right)\;\;+\;\;H^{+}\left(\mathrm{aq}\right)\;\;+\;\;e^{-}\;\;+*\;\;\to \;\;*\mathrm{COOH}$$

(2) $$*\mathrm{COOH}\;\;+\;\;H^{+}\left(\mathrm{aq}\right)\;\;+\;\;e^{-}{}^{}\;\;\to \;\;*\mathrm{CO}\;\;+\;\;H_{2}O$$

(3) ∗CO  →  CO  +∗

(4) $$H^{+}\left(\mathrm{aq}\right)\;\;+\;\;e^{-}\;\;+*\to \;\;*H$$

(5) $$*H\;\;+\;\;H^{+}\left(\mathrm{aq}\right)\;\;+\;\;e^{-}\;\;\to \;\;H_{2}\;\;+*$$

The facilitated stabilization (Equation (1)) and reduction (Equation (2)) of *COOH as well as the fast desorption of CO molecules (Equation (3)) together contributed to the high CO yield. In addition, the HER process (Equations (4) and (5)) as a major side reaction was effectively suppressed. Moreover, on the surface of relatively small Au NPs, the increased low‐coordinated sites and active edge sites could contribute to the stabilization of *COOH intermediates and the production of CO rather than the competitive HER process.^80^ According to the free energy (Δ*G*) diagrams of CO_2_ electroreduction to CO (Figure 4e,f), the overpotential mainly results from the step of COOH* formation. Due to the presence of an optimum ratio of edge sites on tiny‐sized Au NPs (Au38), the energy barrier between CO_2_ and *COOH was decreased, thus could realize a lower overpotential for higher CO yield. Besides, concave rhombic dodecahedron Au NPs (Figure 4g) were synthesized to explore the importance of high‐index planes, such as (332) and (775) facets, achieving good activity and high stability (Figure 4h).^81^

The effects of surface modifications on Au NPs have also been investigated. Feng and co‐workers deposited Au NPs with a relatively high density of grain boundaries on carbon nanotubes (Au/CNTs), which could improve the catalytic activity for CO generation by stabilizing unique active surfaces.^82^ The linear correlation between the reduction activity and the density of grain boundaries indicated the edges of grain boundaries could act as active sites for stronger adsorption of *COOH intermediates. These results provide new insights to control sizes, exposed facets and morphology of metal nanocrystals for improving the performance of CO_2_ reduction.

Ag metal as an appropriate candidate presents outstanding selectivity for CO generation. Ag disk electrodes with the assistance of imidazolium‐based ionic liquids exhibited selective CO production, owing to the immobilization of CO_2_•^−^ intermediates by C4‐ and C5‐protons on imidazolium rings.^83^ Guo et al. prepared bovine serum albumin‐capped Ag nanoclusters demonstrating a high Faradaic efficiency up to 75% for selective CO evolution in dimethylformamide aqueous solution.^84^

**Figure 5:**
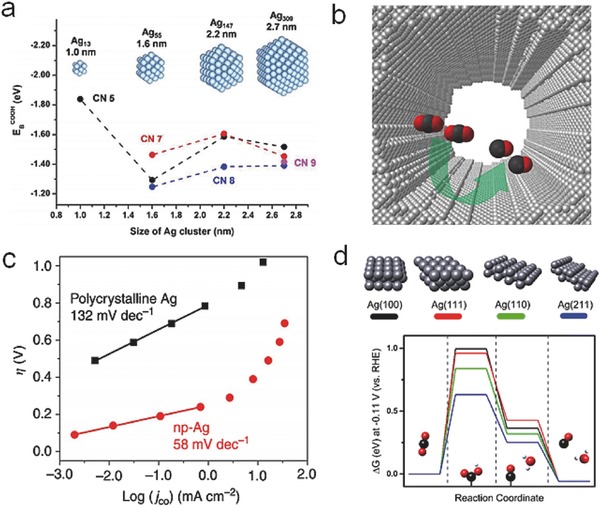
a) DFT calculation results on the binding energies of *COOH intermediates as a function of the size of Ag NPs. Reproduced with permission.^86^ Copyright 2015, American Chemical Society. b) Schematic diagram of nanoporous Ag (scale bar, 500 nm). c) The partial current density of CO production under different overpotentials on polycrystalline silver and nanoporous Ag, respectively. Reproduced with permission.^87^ Copyright 2014, Macmillan Publishers Limited. d) Free energy diagrams for the electroreduction of CO_2_ to CO on flat (Ag(100) and Ag(111)) and edge (Ag(221) and Ag(110)) sites. Reproduced with permission.^88^ Copyright 2015, American Chemical Society.

Carrying out the CO_2_ reduction reaction with a bulk Ag electrode in ion liquid solutions or organic electrolytes is not conducive to large‐scale industrial applications. Luckily, Ag nanostructures with optimized size, structure and surface modification can also realize enhanced properties. Nanosized Ag electrodes possess abundant active sites and can achieve highly selective CO production at an overpotential lower than bulk electrode or flat surface.^85^, ^86^ Since more low‐coordinated atoms exposed on the surface of smaller sized Ag, it could promote the formation of Ag‐COOH bonds to stabilize *COOH intermediates (**Figure** **5**a). Lu et al. prepared nanoporous Ag NPs with highly curved surface (Figure 5b), which could achieve ≈92% of Faradaic yield for CO production.^87^ The strong adsorption of *COO^−^ intermediates on nanoporous Ag can lead to a rapid first‐electron transfer step superior to that on polycrystalline Ag (Figure 5c). The density functional theory (DFT) calculations and experimental tests of CO_2_ reduction on the surface of nanosized Ag were also investigated.^88^ The abundant exposed active edge/corner sites of Ag NPs could decrease the activation energy barrier of electron transfer, thus beneficial for the yield and selectivity of CO production (Figure 5d).

The catalytic activity of Ag nanocatalysts can be further improved by surface modifications. To form an active surface layer on Ag electrode, oxidation–reduction method,^89^ electrochemical deposition,^90^ and anodization treatment^91^ can be utilized. Through surface modifications, the derived Ag electrocatalysts can exhibit higher specific surface area and stronger adsorption of *COOH and *COO^−^ intermediates, thus leading to higher activity and suppression of H_2_ evolution. The modified Ag nanocatalysts can realize a Faradaic yield as high as 90% for CO production at relatively low overpotentials.

**Figure 6:**
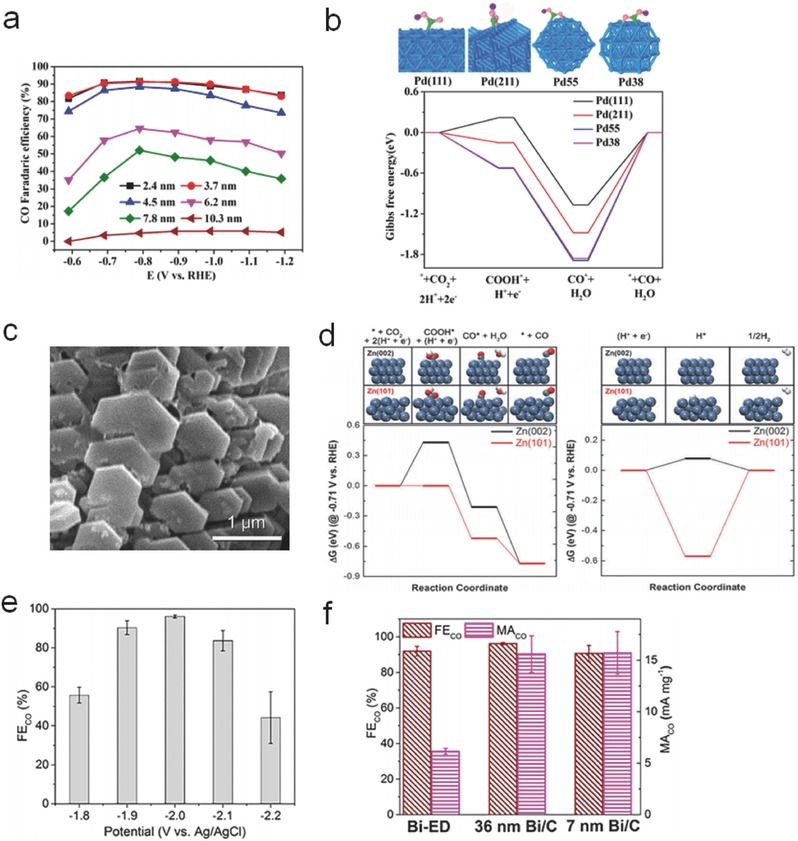
a) Applied potential dependence of Faradaic efficiencies for CO production over Pd NPs with different sizes. b) Adsorption of *COOH (top) and DFT results on the free energy for CO_2_ reduction to CO (bottom) on Pd(111), Pd(211), Pd55, and Pd38. Reproduced with permission.^94^ Copyright 2015, American Chemical Society. c) SEM image of hierarchical hexagonal Zn. d) Free‐energy diagrams of CO_2_ reduction (left) and HER (right) on Zn (002) and Zn (101). Reproduced with permission.^99^ (e) Faradaic efficiencies of CO production under different applied potentials on 36 nm freshly reduced Bi/C. f) Faradaic efficiencies and mass activities of CO production on electrodeposited Bi films (Bi‐ED), 36 or 7 nm freshly reduced Bi/C by hydrazine (36 nm Bi/C or 7 nm Bi/C). Reproduced with permission.^100^ Copyright 2016, American Chemical Society.

Pd‐based electrocatalysts have also been studied for CO_2_ reduction. Owing to the poor catalytic activity of polycrystalline Pd foil,^92^ efforts have been made to construct nanostructural Pd for enhancing the activity and Faradaic yield. Novel Pd/C nanocatalyst^93^ exhibited high mass activities (50–80 mA mg^−1^) for HCOO^−^ generation due to the formation of PdH*_x_* through a rapid electrohydrogenation step. Gao et al. explored the size‐dependent electrocatalytic activity of Pd/C NPs for generating CO,^94^ showing high Faradaic efficiency up to 91.2% at −0.89 V (vs RHE) using 3.7 nm Pd NPs, which was comparable to that of Au or Ag (**Figure** **6**a). The ratios of corner, edge, or terrace active sites can be modified by different sizes and morphologies of Pd NPs. According to the DFT calculation results (Gibbs free energy diagrams in Figure 6b), the steps of CO_2_ adsorption, *COOH formation, and *CO removal prefer to occur on smaller Pd NPs with a higher ratio of corner and edge sites. However, compared to Au and Ag metals, Pd NPs would be more easily deactivated by the poisoning of adsorbed CO after a period of reaction time at excessive overpotential.

Zn, as a low‐cost and earth‐abundant metal, has been regarded as a promising electrocatalyst with high selectivity of CO production. However, the stability and catalytic activity are the predominant obstacles to be overcome. Previously, Hattori and co‐workers have explored bulk Zn electrocatalyst, which can successfully convert CO_2_ molecules into CO with a considerable current density.^95^, ^96^ Nevertheless, the efficiency of bulk Zn is hard to be improved due to the rapid oxidation on the surface. Nanosized Zn, such as nanostructured Zn dendrites synthesized by an electrodeposition approach,^97^ presented higher activities than bulk ones, owing to the minimization of surface oxide layer. With the assistance of NaCl electrolyte, nanoscale Zn could generate CO with a Faradaic efficiency of 93% since the adsorption of Cl^−^ ions on Zn surface is conducive to the formation of *COO^−^ intermediates.^98^ However, the stability was not satisfying due to the inevitable oxidization during electrolysis. Recently, hierarchical hexagonal Zn (Figure 6c) showed a Faradaic efficiency of 85.4% for selective CO production over 30 h.^99^ DFT calculation revealed that the exposed Zn (101) facet favored the stabilization of *COOH intermediates (Figure 6d). The researches indicated that superior catalytic performances could be achieved by the design of morphologic structure, especially exposed facets.

Due to earth‐abundant, cheap and pollution‐free features, Bi metal also has been applied for reducing CO_2_ to CO. The Bi NPs activated through hydrazine treatment performed the highest Faradaic yield of CO production (96.1%) in acetonitrile based electrolyte (Figure 6e,f).^100^ In another case, nanostructured Bi nanoflakes were directly grown on Cu film by a pulse electrodeposition method,^101^ showing a large number of edge/corner sites and unexpected Faradaic efficiency of 79.5% for HCOO^−^ generation.

#### 2.2.3 Cu

Cu as the third group metal can generate high value‐added carbonaceous compounds at low cost.^102^, ^103^, ^104^ However, poor selectivity and activity degradation are two remaining challenges for practical application. Great efforts are still needed to reduce the overpotential, optimize the selectivity, and stability.^105^, ^106^ Many experimental factors such as morphology, surface modification, crystal planes, and active sites can lead to different reaction pathways and various products.

**Figure 7:**
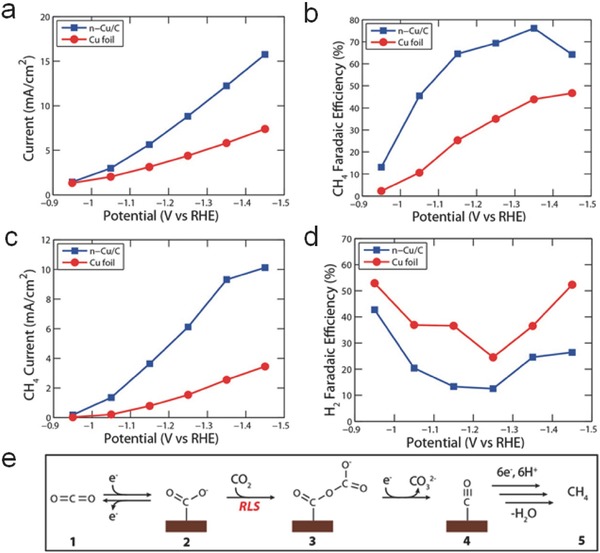
Comparison of current densities and Faradaic efficiencies of *n*‐Cu/C and copper foil. a) Total current density of *n*‐Cu/C and copper foil. b) Faradaic efficiencies for CH_4_ generation. c) Methanation current densities. d) Faradaic efficiencies for H_2_ generation, showing suppressed H_2_ evolution on *n*‐Cu/C catalyst. e) Proposed mechanism for the electrochemical reduction of CO_2_ to CH_4_, including the rate‐limiting step (RLS), consistent with the electrochemical data and known intermediates identified in the literature. Reproduced with permission.^110^ Copyright 2014, American Chemical Society.

Diverse morphologies (such as NPs, nanowires, nanocubes, etc.) of Cu nanocrystals have been investigated for the generation of HCOO^−^, CO, hydrocarbons, and alcohols. Cu nanopillars exhibited a Faradaic efficiency of 28% for the yield of HCOOH at −0.5 V (vs RHE).^107^ Porous hollow Cu fibers^108^ and Cu nanowires^109^ were employed to achieve distinct electrocatalytic selectivity of CO at low overpotentials. Cu NPs loaded on glassy carbon (*n*‐Cu/C) achieved a Faradaic efficiency up to ≈80% for CH_4_ generation, with four‐times higher energy efficiency than Cu foil (**Figure** **7**a–d).^110^ As displayed in Figure 7e, a detailed reaction mechanism of CO_2_ reduction using *n*‐Cu/C was described. The *CO_2_^−^ intermediates formed by a one electron‐transfer pre‐equilibrium step were strongly absorbed on the active surface of Cu. Then, *CO_2_^−^ reacted with another CO_2_ molecule to yield a *CO_2_–CO_2_^−^ intermediate by C−O coupling, realizing CH_4_ production through the formation and further reduction/hydrogenation of *CO intermediates. By utilizing different nanostructured Cu, like NPs,^111^ nanocubes,^112^ and nanowires,^113^ other hydrocarbons and alcohols (such as ethylene, ethane, *n*‐propanol, and ethanol) could generate through CO dimerization pathways (Figure 3).

**Figure 8:**
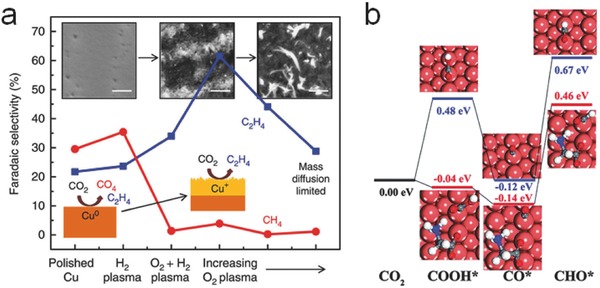
a) Hydrocarbon selectivity of plasma‐treated Cu foils. Reproduced with permission.^114^ Copyright 2016, the Author, published under CC‐BY 4.0 license. b) The DFT calculated free energy change of CO_2_ and CO protonation without glycine (blue lines) and with glycine (red lines). Reproduced with permission.^116^ Copyright 2016, The Royal Society of Chemistry.

Surface modification of Cu electrodes has also been investigated in recent years. To achieve high selectivity toward C_2_ hydrocarbon products (such as C_2_H_4_ and C_2_H_5_OH), oxygen plasma‐activated Cu^114^ and oxygen‐derived Cu mesoporous foam^115^ have shown high Faradaic efficiency. With an increased local pH value on the oxidized Cu surfaces, the CO dimerization would be promoted, which is beneficial to the yield of ethylene, as illustrated in **Figure** **8**a. Cu nanowires modified by amino acid were also tested (Figure 8b).^116^ The results confirmed that the introduced —NH_3_^+^ group on Cu surface can benefit the stabilization of *CHO intermediates and the subsequent hydrogenation reaction for producing C_2_ and C_3_ hydrocarbons.

**Figure 9:**
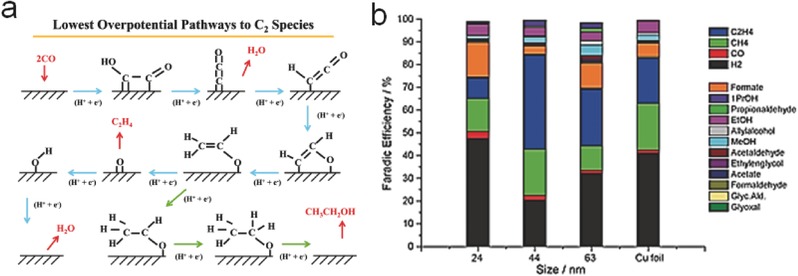
a) Schematic illustration of the species involved in the reaction pathways to generate C_2_H_4_ (blue) and C_2_H_5_OH (green). Reproduced with permission.^118^ b) Bar graph reporting the Faradaic efficiencies for each product produced by Cu foil and Cu nanocubes with different sizes at −1.1 V versus RHE. The glassy carbon signal has been subtracted. Reproduced with permission.^119^

Previous works have discovered some intimate relationships between product selectivity and exposed lattice planes or active sites of Cu electrodes. Cu nanocatalysts with different exposed crystal facets (Cu (111), Cu (211), and Cu (100)) could lead to multiple products under the same condition.^112^, ^117^ Densely packed (111) facets preferred to generated HCOOH, while highly stepped (211) facets was superior for CH_4_ generation. Close‐packed (100) facets performed the most favorable selectivity for C_2_ hydrocarbons instead of C_1_ products, by means of a sequential electron–proton transfer and the reduction of ethylene oxide (C_2_H_3_O) intermediates, as illustrated in **Figure** **9**a.^118^ Moreover, an optimal ratio of edge sites over (100) planes played a crucial role toward CO_2_ reduction and C_2_H_4_ production, as verified by the experimental results using Cu nanocubes of different sizes (Figure 9b).^119^ Therefore, it is meaningful to utilize Cu as a model electrocatalyst for the studies of CO_2_ reduction.

### 2.3 Metal Alloys

Metal alloys can enhance the electrocatalytic reaction kinetics and selectivity of CO_2_ reduction by adjust the binding capability of active intermediates (such as *COOH and *CO). For example, a novel Pd*_x_*Pt_(100−_ *_x_*_)_/C electrocatalyst was reported to convert CO_2_ into HCOOH at ≈0 V (vs RHE), which considerably approached the theoretical equilibrium potential of 0.02 V (vs RHE).^120^ However, the high cost and low stability of noble metals still need to be resolved.

**Figure 10:**
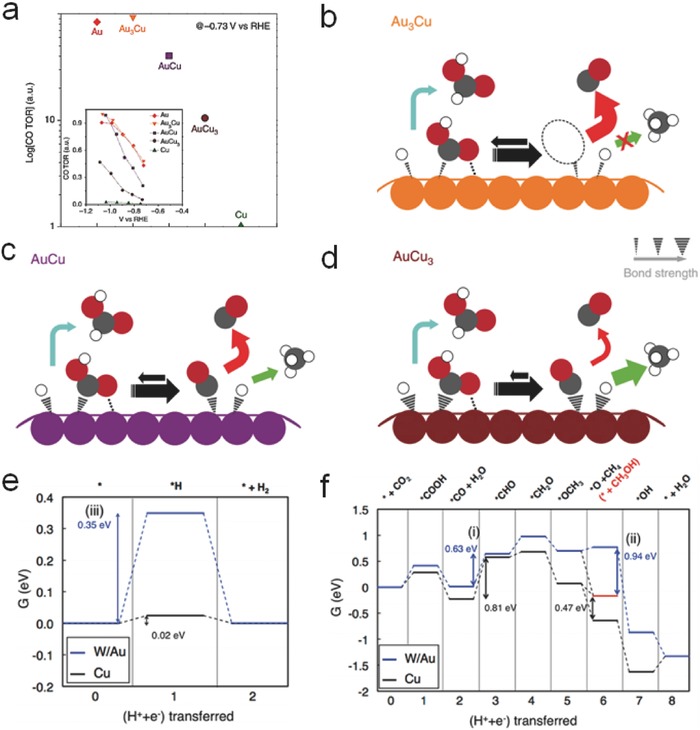
a) Relative turnover rates (TORs) for CO generation and (b–d) proposed mechanism for CO_2_ reduction on the Au–Cu bimetallic NPs. Reproduced with permission.^121^ Copyright 2014, Macmillan Publishers Limited. Free energy diagrams for e) H_2_ evolution and f) CO_2_ electroreduction to CH_4_ or CH_3_OH on W/Au and Cu electrodes. Reproduced with permission.^125^ Copyright 2014, American Chemical Society.

The introduction of nonnoble‐metals into the alloy electrocatalysts can minimize the cost and improve the performances. Recently, some reports have investigated the activity of Cu alloys. Compared to Au or Cu NPs, nanosized Au_3_Cu alloys assembled into ordered monolayers^121^ showed higher Faradaic efficiency for CO production (**Figure** **10**a). Both the electronic effect and geometric effect of Au*_m_*Cu*_n_* alloys should be taken into consideration for the selective CO production and the desorption ability of *COOH. The higher d‐band levels of Cu can enhance the binding capability of *COOH and *CO, which is conducive to the production of hydrocarbons. However, when referred to the geometric effect, Cu atoms next to the Au—C bonds can further stabilize *COOH and lead to the generation of CO (Figure 10b–d). Therefore, an appropriate content of Cu in Au–Cu alloys can promote CO production. Rasul et al. developed a Cu–In alloy electrocatalyst,^122^ which could selectively convert CO_2_ into CO with a Faradaic efficiency of 95% as well as negligible H_2_ or HCOOH evolution. It is because the intact Cu corner sites and the surface of In both promote a strong binding capability of *COO^−^ superior to that of *H intermediates. Recently, a Cu–Sn bimetallic electrode achieved a high Faradaic efficiency over 90% for CO productivity by introducing an optimal amount of Sn.^123^ As the ratio of Sn atoms increased, the multifold sites on Cu were disturbed, thus inhibited the adsorption of *H on catalyst surface.

Some high‐value hydrocarbon compounds can be generated using other metal alloys. Torelli et al. prepared Ni–Ga films for the production of methane, ethylene, and ethane.^124^ The *COOH intermediates could be tightly bound on the surface of Ni and the introduction of Ga can weaken the Ni–CO interaction, therefore Ni and Ga synergistically increased the yields of C_2_ hydrocarbons and avoided the poisoning of CO on the catalyst surface. Analyzed by a computational calculation method (Figure 10e,f), W–Au alloy was regarded as a suitable candidate to decrease the overpotential for *COO^−^ formation and suppress unfavorable *H adsorption for methanol production,^125^ possibly followed a pathway: CO_2_ → *COO^−^ → CO_ads_ → CHO_ads_ → CH_3_O_ads_ → methanol. Sun et al. developed a Mo–Bi bimetallic electrocatalyst with high CH_3_OH selectivity, which achieved a maximum Faradaic efficiency of 71.2% in acetonitrile with the assistance of ion liquids.^126^ In brief, these works exhibited the possibility to realize low‐cost and highly active alloy electrocatalysts for the scalable CO_2_ electroreduction.

### 2.4 Inorganic Metal Compounds

#### 2.4.1 Metal Oxides

Metal‐oxide‐based electrocatalysts have gradually got attention due to their decent energy efficiency and selectivity for CO_2_ electroreduction, although the instability is still a big problem.

According to DFT calculation results, HCOOH, methane, or methanol could be produced at different conditions with RuO_2_ electrocatalyst by adjusting the *CO coverage.^127^ Pb_2_O cathode exhibited a Faradaic efficiency of 60% for HCOOH generation in KHCO_3_ aqueous solution.^128^ However, owing to the high cost or toxicity, it is impracticable to used RuO_2_ or Pb_2_O for CO_2_ reduction. Instead, other earth‐abundant and low‐toxicity metals, such as Sn, Co, Ni, and Ti, have been considered as alternative electrocatalysts.

**Figure 11:**
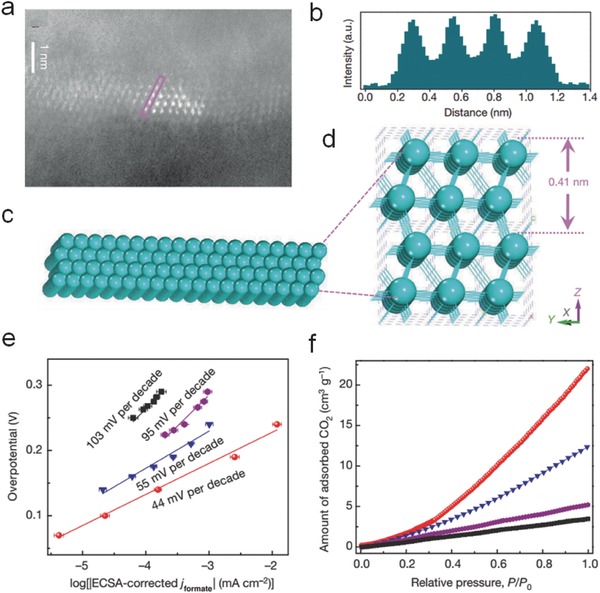
a) Lateral high‐angle annular dark‐field scanning transmission electron microscopy (HAADF‐STEM) image of partially oxidized Co 4‐atom‐thick layers and (b) the corresponding intensity profile along the pink rectangle in (a). c,d) Corresponding crystal structures. e) Electrochemical active surface area (ECSA) corrected Tafel plots for HCOO^−^ production. f) CO_2_ adsorption isotherms of partially oxidized Co 4‐atom‐thick layers (red), Co 4‐atom‐thick layers (blue), partially oxidized bulk Co (violet) and bulk Co (black). Reproduced with permission.^131^ Copyright 2016, Macmillan Publishers Limited.

SnO*_x_*/CNT cathodes performed a 60% Faradaic efficiency with 25% energy efficiency for HCOOH formation.^129^ Xie and co‐workers synthesized ultrathin Co_3_O_4_ layers with 1.72 nm thickness as an effective electrocatalyst, showing an optimum 64.3% Faradaic efficiency for HCOO^−^ production after 20 h reaction.^130^ Later, the same group prepared partially oxidized Co 4‐atomic‐layers with an average thickness of 0.84 nm (**Figure** **11**a–d), and achieved a ultrahigh HCOO^−^ selectivity of 90.1% over 40 h.^131^ The Co based atomic layers possessed abundant active sites, and the increased charge density near Fermi level could improve electronic conductivity. The process of HCOO^−^ production was occurred as below

(6) $${\mathrm{CO}}_{2}\left(g\right)\;\;+*\;\;\to \;\;*{\mathrm{CO}}_{2}$$

(7) $$*{\mathrm{CO}}_{2}\;\;+\;\;e^{-}\;\;\to \;\;*{\mathrm{COO}}^{-}$$

(8) $$*{\mathrm{COO}}^{-}\;\;+\;\;H^{+}\;\;+\;\;e^{-}\;\;\to \;\;*{\mathrm{HCOO}}^{-}$$

(9) $$*{\mathrm{HCOO}}^{-}\;\;\to \;\;{\mathrm{HCOO}}^{-}\;\;+*$$

The Tafel slopes (≈59 mV dec^−1^, Figure 11e) and preferable CO_2_ adsorption capability (Figure 11f) of partially oxidized Co 4‐atomic‐layers indicated the good properties for CO_2_ activation and *COO^−^ intermediate stabilization.

Some other metal oxides were also investigated. NiO showed a Faradaic efficiency up to 35.2% for syngas (CO and H_2_) products.^132^ Nanostructured TiO_2_ films were applied for CO_2_ reduction in acetonitrile electrolyte.^133^ The oxygen vacancies (or Ti^3+^ species) on TiO_2_ films were identified as active sites, which could strongly bind CO_2_ molecules and promote the generation of *COO^−^ intermediates, resulting in the generation of methanol as primary product. These results indicated that the introduction of oxidation states in certain metallic catalysts can greatly improve the performance for CO_2_ electrochemical reduction.

#### 2.4.2 Metal Chalcogenides

Interestingly, some chalcogenides of transition metals (such as Fe, Mo, W) were found to be available catalysts for electrocatalytic CO_2_ reduction. The reaction intermediates can be bound to different active sites on the surface of metal chalcogenides, therefore the limitation of linear‐scale relations between the binding energies of reaction intermediates and specific metals can be broken. It is also worth noting that the different edge sites of metal chalcogenides can perform different duties for the generation of varied products. In 2011, porous ternary chalcogels of Ni–Fe_4_S_4_ and Co–Fe_4_S_4_ with high surface area and high charge mobility were applied to improve the electrocatalytic activity for CO and CH_4_ production.^134^

**Figure 12:**
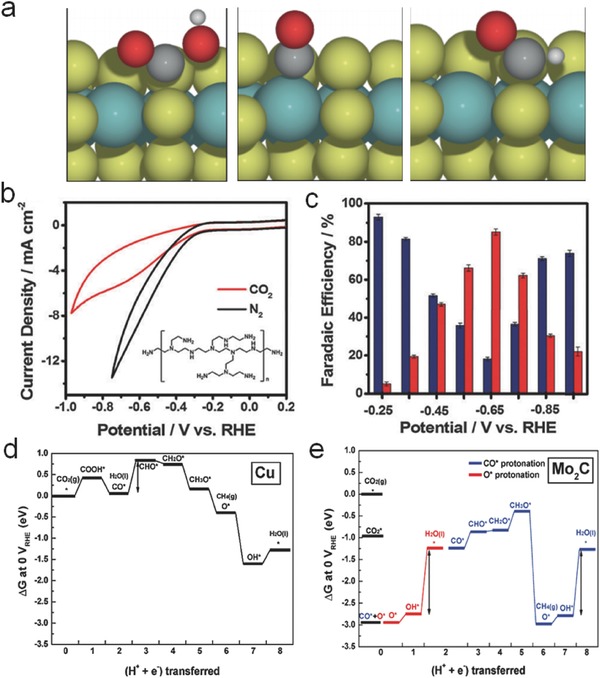
a) Binding configurations of *COOH, *CO, and *CHO on the Mo edge of MoS_2_. *COOH and *CHO preferably bind to the bridging S atoms, while *CO binds to the Mo atoms. Reproduced with permission.^135^ b) Cyclic voltammograms (CVs) of rGO–PEI–MoS*_x_* modified glassy carbon electrode in N_2_‐saturated and CO_2_‐saturated 0.5 m aqueous NaHCO_3_ solution, respectively. Inset: Structure of PEI. c) Faradaic efficiency for CO (red bars) and H_2_ (blue bars) production at different applied potentials. Reproduced with permission.^136^ Copyright 2016, The Royal Society of Chemistry. Free energy diagrams of CO_2_ conversion to CH_4_ over d) Cu (211) and e) Mo_2_C (100) surfaces at 0 V (vs RHE), respectively. The most endergonic step in the overall process is designated with an arrow. Reproduced with permission.^141^ Copyright 2016, American Chemical Society.

Nørskov and co‐workers investigated the active edge sites of MoS_2_, MoSe_2_, and Ni‐doped MoS_2_ (Ni–MoS_2_) simulated by DFT method.^135^ The *COOH and *CHO intermediates prefer to attach to bridging S or Se atoms, while *CO intermediates trend to bind with the edge sites of metal atoms (**Figure** **12**a). All edges were involved in CO evolution, while the S edges of Ni–MoS_2_ and the Mo edges of MoSe_2_ could further turn CO to hydrocarbons or alcohols. Inspired by this, a cost‐effective MoS*_x_* electrocatalyst was introduced, which can produce syngas (CO and H_2_) at a low overpotential of ≈290 mV and achieve a maximum Faradaic efficiency of 85.1% for CO yield with the assistance of reduced graphene oxide (rGO) and polyethylenimine (PEI) (Figure 12b,c).^136^ In 2016, Nørskov and co‐workers found that the combination of dopant metal sites (*CO binding sites) and S binding sites (*COOH, *CHO, and *COH binding sites) on metal‐doped MoS_2_ can provide two different linear‐scaling relationships, which synergistically result in enhanced CO_2_ reduction performance than pristine Mo metal.^137^ The as‐prepared Co‐doped MoS_2_ could achieve better electrocatalytic ability for methanol production than pristine MoS_2_.^138^

Compared to MoS_2_ and MoSe_2_, WSe_2_ has the lowest work function that can facilitate rapid electron transfer during CO_2_ reduction. Hence, WSe_2_ was considered as another promising candidate for CO production with high current density, which can perform a current density 60 times higher than that of Ag NPs under the same condition.^139^ Thanks to the low work function and high d‐electron density on the W‐terminated edge sites, the *COOH and *CO intermediates were more stable on WSe_2_ than Ag, resulting in the easier formation of CO on WSe_2_ at low overpotentials. We expect that further efforts devoted to metal chalcogenides will be very helpful to the research of carbon fixation in the future.

#### 2.4.3 Metal Carbides

Transition metal carbides are another class of promising catalysts with low cost, favorable carbophobic and oxophilic properties. In 2015, to understand the relationships between the binding energies of reaction intermediates and active sites of metal carbides, Wannakao et al. studied the CO_2_ reduction mechanism of Fe‐, Co‐, and Pt‐doped W carbides through DFT method.^140^ The results showed that the d‐band center of transition metal was related to the adsorption energies, which relatively influenced the binding site preferences and geometries of the active intermediates. Therefore, the electron structure of W carbide catalysts could be tuned by metal doping to improve the carbophilicity and oxophilicity for the optimized activity and selectivity.

Kim et al. reported that Mo_2_C could convert CO_2_ into CH_4_ at an onset potential of −0.55 V (vs RHE), of which free energetics of CO hydrogenation was less than that of conventional Cu metal.^141^ The DFT‐calculated free energies for CO_2_ reduction to CH_4_ on Cu(211) surface are displayed in Figure 12d, which shows that the major energy‐consuming step for CH_4_ generation is the protonation of adsorbed *CO (−0.74 V vs RHE). In contrast, CO_2_ molecules were preferably adsorbed on Mo_2_C (100) surface (Figure 12), followed by the dissociation of C—O bonds at the initial reaction stage before protonation. Once the *O intermediates were generated by the C—O bond fission, the protonation was easily accessible because of a lower potential demand (about −0.20 V vs RHE). Hence, other than Cu, the rate‐limiting factors on Mo_2_C surface for selective CH_4_ production were determined to be the *OH removal and the nonelectrochemical C—O bond scission. The new insights on metal oxides/chalcogenides/carbides opened a new field for the design of low cost catalysts and new theoretical foundation for electrochemical CO_2_ reduction.

### 2.5 Carbon‐Based Metal‐Free Electrocatalysts

Metals like Pd, Au, Ag, and Cu have been popularly employed as electrocatalysts for CO_2_ reduction. However, there are some issues to be resolved, such as relatively costly price, high overpotential, and inferior selectivity. To address these problems, carbon‐based nanomaterials like carbon nanotubes, graphene, carbon fibers, and porous carbon have been considered as potential alternatives, which could bring about decent activity and low cost. However, when compared to the field of oxygen reduction reaction and water splitting, the relevant investigations of carbon‐based electrocatalysts for CO_2_ reduction are still quite few.

**Figure 13:**
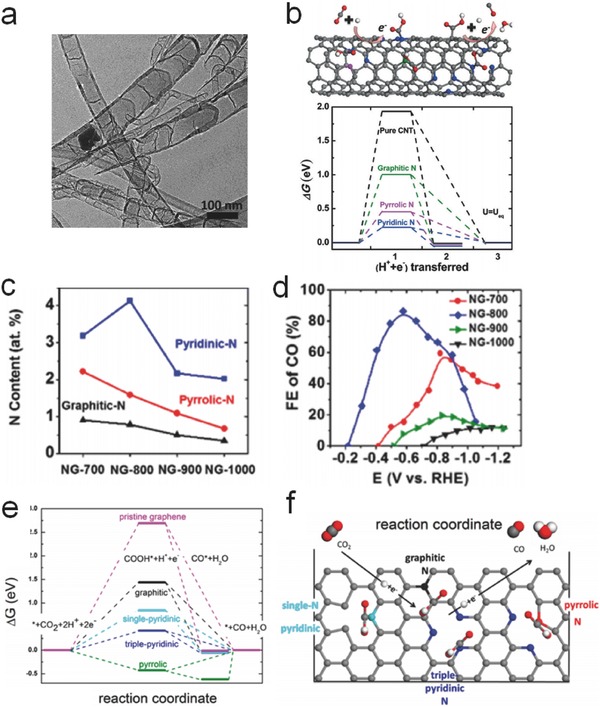
a) TEM image of bamboo‐shaped NCNTs. b) Schematic of CO formation on NCNTs and free‐energy diagram at equilibrium potential for CO_2_ reduction on pyridinic‐N, pyrrolic‐N, and graphitic‐N defects compared to original CNTs. Reproduced with permission.^144^ c) The corresponding N functionality content and d) Faradaic efficiency of CO production versus applied potential on N‐doped graphene with different doping temperatures (700–1000 °C). e) Free energy diagrams of electrocatalytic CO_2_ conversion on N‐doped graphene and f) schematic of nitrogen defects and CO_2_ reduction mechanism. Reproduced with permission.^150^ Copyright 2015, American Chemical Society.

Nitrogen‐doped CNTs (NCNTs) could realize effective CO_2_ capture and high product selectivity for CO generation at a significantly decreased overpotential than pristine CNTs.^142^, ^143^ Compared to pristine CNTs, the introduction of pyridinic‐N into bamboo‐shaped NCNTs (**Figure** **13**a) led to higher electrical conductivity and achieved a Faradaic efficiency of 80% for CO generation.^144^ Among the different N defects (Figure 13b), pyridinic‐N sites exhibited the highest binding capability with CO_2_ molecules and the lowest absolute overpotential (0.20 V) for *COOH formation, which promoted the CO formation. Other than pyridinic‐N, the existence of quaternary‐N or pyrrolic‐N could stabilize the radical active intermediates and lower the reduction barriers.^145^ Moreover, with the help of PEI cocatalyst, NCNTs were employed for converting CO_2_ into HCOO^−^ with an overpotential of ≈0.54 V (vs NHE), attributing to the strong stabilization of the *COO^−^ intermediates.^146^

N‐doped graphene has also been investigated for CO and HCOO^−^ 147, 148 as well as CH_4_.^149^ The pyridinic‐N or pyrrolic‐N species resulted in stronger CO_2_ adsorption and lower energy barrier for the formation of *COOH intermediates. Similarly, the incorporation of pyridinic‐N defects into 3D graphene foam can also lower the free energy barrier to form adsorbed *COOH and facilitate the CO yield (Figure 13c,d).^150^ The corresponding free energy diagrams for selective CO generation on different sites of N‐doped graphene and pristine graphene through the lowest energy‐consuming pathway are explicitly shown in Figure 13e. The excess overpotential is resulted from the uphill barrier of the first electron‐transfer rate‐determine step for *COOH formation. The *COOH intermediates have good affinity with N defects, and the free energy barrier for *COOH adsorption decreases significantly on pyridinic‐ or pyrrolic‐N sites rather than graphitic‐N sites (Figure 13f). Subsequently, the second proton‐coupled electron transfer becomes thermodynamically easier for the formation of adsorbed *CO (or CO). Interestingly, it was found that boron‐doped graphene can efficiently generate the exclusive product of HCOO^−^ at low overpotentials.^151^ GO/CNTs composite has also been reported for converting CO_2_ to CO, showing higher selectivity and activity than noble metals (Au and Ag).^152^ However, the need of adding ionic liquids makes it difficult to be used for large‐scale applications.

Some other carbon‐based materials, such as carbon fibers,^153^ metal‐doped nitrogenated carbon black,^154^ N‐doped diamond,^155^ nanoporous carbon,^156^ B‐doped diamond,^157^ and Cu NPs/B‐doped diamond^158^ were found can achieve CO, CH_4_, HCOO^−^, or HCHO formation with high selectivity and low overpotential due to their preferable adsorption of CO_2_ and suitable binding capability of active intermediates. The study of metal‐free carbon‐based electrocatalysts has opened a new door for developing cheaper alternatives instead of precious noble metals.

## 3 Product Selectivity in Electrocatalytic CO_2_ Reduction

The electrocatalysts play an important role to the product selectivity of CO_2_ reduction. Different electrocatalysts have shown diverse tendencies of generating specific carbonaceous compounds, such as HCOOH/HCOO^−^, CO, formaldehyde (HCHO), hydrocarbons, and alcohols, with almost unavoidable H_2_ evolution as side reaction. However, the product selectivity of CO_2_ reduction seems quite complicated and closely related to the reaction conditions and pathways. Not only the electrocatalyst, the intricate reaction steps can also be influenced by many other parameters, such as applied potential, electrolyte, pH value, temperature, and pressure. To ensure our readers can quickly find valuable recapitulative data from related literatures, **Table** **2** summarized a series of representative experimental results obtained from different electrocatalysts and reaction conditions (electrolytes and applied potentials), together with the associated information of measured selectivities and activities.

**Table 2:** The representative examples of electrochemical CO_2_ reduction with different electrocatalysts, reaction conditions and selectivities

Electrocatalyst	Electrolyte	Applied potential [V]	Major products [Faradaic efficiency, %]	Current density/mass activity	Ref.
1. Selective production of HCOO^−^/HCOOH					
[Cu(cyclam)](ClO_4_)_2_ complex	DMF/H_2_O (97:3 v/v)	−2.0 (vs Fc/Fc^+^)	HCOOH (90%)	1 mA cm^−2^	41
Gas‐diffusion layer/CNT/Ir complex/polyethylene glycol	0.5 m LiClO_4_/0.1 m NaHCO_3_/1% v/v MeCN	−1.40 (vs RHE)	HCOO^−^ (83%)	15.6 mA cm^−2^	54
Pd‐polyaniline/CNTs	0.1 m KHCO_3_	−0.80 (vs SCE)	HCOO^−^ (83%)	–	57
SnO*_x_*/Sn	0.1 m KHCO_3_	−1.36 (vs RHE)	HCOO^−^ (71.6%)	17.1 mA cm^−2^	71
Nanostructured Sn	0.1 m NaHCO_3_	−1.80 (vs SCE)	HCOO^−^ (93.6%)	10.2 mA cm^−2^	72
Sn	Ion liquids/H_2_O/MeCN	−2.30 (vs Ag/AgCl)	HCOOH (92.0%)	32.1 mA cm^−2^	77
Pb			HCOOH (91.6%)	37.6 mA cm^−2^	
Pd NPs	0.5 m NaHCO_3_	−0.35 (vs RHE)	HCOO^−^ (88%)	3.45 mA cm^−2^	93
Bi nanoflakes	0.1 m KHCO_3_	−0.40 (vs RHE)	HCOO^−^ (79.5%)	–	101
Bi/BiOCl	0.5 m KHCO_3_	−1.50 (vs SCE)	HCOO^−^ (≈92%)	3.7 mA mg^−1^	161
Cu pillars	0.1 m KHCO_3_	−0.50 (vs RHE)	HCOOH (28.7%)	≈1.3 mA cm^−2^	107
Cu nanofoam	0.5 m KHCO_3_	−1.50 (vs Ag/AgCl)	HCOOH (37%)	–	162
Ag–Sn alloy	0.5 m NaHCO_3_	−0.80 (vs RHE)	HCOOH (≈80%)	≈16 mA cm^−2^	165
Pd*_x_*Pt_(100−_ *_x_*_)_/C	0.1 m KH_2_PO_4_/0.1 m K_2_HPO_4_	−0.40 (vs RHE)	HCOOH (88%)	≈5 mA cm^−2^	120
SnO_2_ porous nanowires	0.1 m KHCO_3_	−0.80 (vs RHE)	HCOO^−^ (80%)	(−1.0 V) 10 mA cm^−2^	163
Mesoporous SnO_2_ nanosheets/carbon paper	0.5 m NaHCO_3_	−1.60 (vs Ag/AgCl)	HCOO^−^ (≈87%)	50 mA cm^−2^	164
Pb_2_O	0.5 m KHCO_3_/NaHCO_3_	−2.0 (vs Co_3_O_4_)	HCOOH (60%/50%)	–	128
Co_3_O_4_ atomic layers	0.1 m KHCO_3_	−0.88 (vs SCE)	HCOO^−^ (64.3%)	0.68 mA cm^−2^	130
Partially oxidized Co atomic layers	0.1 m Na_2_SO_4_	−0.85 (vs RHE)	HCOO^−^ (90.1%)	10.59 mA cm^−2^	131
PEI‐NCNTs/glassy carbon	0.1 m KHCO_3_	−1.80 (vs SCE)	HCOO^−^ (85%)	7.2 mA cm^−2^	146
N‐doped graphene/carbon paper	0.5 m KHCO_3_	−0.84 (vs RHE)	HCOO^−^ (73%)	7.5 mA cm^−2^	148
Boron‐doped graphene	0.1 m KHCO_3_	−1.40 (vs SCE)	HCOO^−^ (66%)	2 mA cm^−2^	151
N‐doped nanodiamond/Si	0.5 m NaHCO_3_	−1.0 (vs RHE)	HCOO^−^ (13.6%) CH_3_COO^−^ (77.6%)	0.75 mA cm^−2^	155
2. Selective production of CO					
Fe TDHPP	DMF/2 m H_2_O	−1.16 (vs RHE)	CO (94%)	0.31 mA cm^−2^	39
Co protoporphyrin–pyrolytic graphite	Perchlorate solution (pH = 3)	−0.60 (vs RHE)	CO (60%)	0.08 mA cm^−2^	40
COF‐366‐Co	0.5 m KHCO_3_	−0.67 (vs RHE)	CO (90%)	80 mA mg^−1^ (Co)	201
[Ru(‐6,6′‐dimesityl‐2,2′‐bipyridine)(CO)_2_Cl]^0^	0.1 m TBAPF_6_/MeCN	≈−2.2 V (vs Fc/Fc^+^)	CO (95%)	–	51
Au‐1,3‐bis(2,4,6‐trimethylphenyl)imidazol‐2‐ylidene complex	0.1 m KHCO_3_	−0.57 (vs RHE)	CO (83%)	≈2 mA cm^−2^	56
Au NPs	0.5 m KHCO_3_	−0.67 (vs RHE)	CO (90%)	–	79
Au rhombic dodecahedrons	0.5 m KHCO_3_	−0.57 (vs RHE)	CO (93%)	–	81
Au/CNTs	0.5 m NaHCO_3_	−0.50 (vs RHE)	CO (≈94%)	≈15 A g^−1^ (Au)	82
Au nanowires	0.5 m KHCO_3_	−0.35 (vs RHE)	CO (94%)	1.84 A g^−1^ (Au)	198
Oxide‐derived Au	0.5 m NaHCO_3_	−0.35 (vs RHE)	CO (>96%)	2–4 mA cm^−2^	199
6 µm thick highly porous Ag	0.5 m KHCO_3_	−0.50 (vs RHE)	CO (82%)	10.5 mA cm^−2^	85
Ag NPs	0.5 m KHCO_3_	−0.75 (vs RHE)	CO (79.2%)	1 mA cm^−2^	86
Nanoporous Ag	0.5 m KHCO_3_	−0.60 (vs RHE)	CO (≈92%)	≈18 mA cm^−2^	87
Ag nanocorals	0.1 m KHCO_3_	−0.60 (vs RHE)	CO (95%)	6.62 mA cm^−2^	89
Oxide‐derived Ag	0.1 m KHCO_3_	−0.80 (vs RHE)	CO (89%)	1.15 mA cm^−2^	200
Pd NPs	0.1 m KHCO_3_	−0.89 (vs RHE)	CO (91.2%)	23.9 A g^−1^ (Pd)	94
Pd icosahedra/C	0.1 m KHCO_3_	−0.80 (vs RHE)	CO (91.1%)	–	166
Zn dendrites	0.5 m NaHCO_3_	−1.10 (vs RHE)	CO (79%)	–	97
Zn foil	0.5 m NaCl	−1.60 (vs SCE)	CO (93%)	–	98
Hexagonal Zn	0.5 m KHCO_3_	−0.95 (vs RHE)	CO (85.4%)	9.5 mA cm^−2^	99
Surface activated Bi NPs	MeCN/[bmim][OTf]	−2.0 (vs Ag/AgCl)	CO (96.1%)	15.6 mA mg^−1^ (Bi)	100
Cu fibers	0.3 m KHCO_3_	−0.40 (vs RHE)	CO (75%)	≈9 mA cm^−2^	108
Cu nanowires	0.1 m KHCO_3_	−0.40 (vs RHE)	CO (61.8%)	1 mA cm^−2^	109
Au_3_Cu alloy	0.1 m KHCO_3_	−0.73 (vs RHE)	CO (64.7%)	3 mA cm^−2^	121
Ordered AuCu NPs	0.1 m KHCO_3_	−0.77 (vs RHE)	CO (80%)	–	167
Cu–In alloy	0.1 m KHCO_3_	−0.60 (vs RHE)	CO (85%)	≈0.75 mA cm^−2^	122
Cu–Sn alloy	0.1 m KHCO_3_	−0.60 (vs RHE)	CO (>90%)	1 mA cm^−2^	123
Oxide‐derived Cu			CO (63%)	2.1 mA cm^−2^	
TiO_2_ film	MeCN/0.1 m TEAP	−1.8 (vs Ag/AgCl)	CO (90%)	–	133
rGO–PEI–MoS*_x_*	0.5 m NaHCO_3_	−0.65 (vs RHE)	CO (85.1%)	55 mA cm^−2^	136
WSe_2_ nanoflakes	50 vol%/50 vol% EMIMBF_4_/H_2_O	−0.164 (vs RHE)	CO (24%)	18.95 mA cm^−2^	139
MoSeS alloy monolayers	4 mol%/96 mol% EMIMBF_4_/H_2_O	−1.15 (vs RHE)	CO (45.2%)	43 mA cm^−2^	168
NCNTs	0.1 m KHCO_3_	−1.05 (vs RHE)	CO (80%)	–	144
N‐doped graphene foam	0.1 m KHCO_3_	−0.58 (vs RHE)	CO (≈85%)	≈1.8 mA cm^−2^	150
3. Selective production of HCHO					
Boron‐doped diamond	MeOH electrolyte	−1.70 (vs Ag/AgCl)	HCHO (74%)	97.5 µA cm^−2^	157
Cu NPs/boron‐doped diamond	(10 × 10^−6^ m) H_2_O/bmim‐PF_6_	−1.3 (vs RHE)	HCOOH and HCHO (>80%)	5.1 mA cm^−2^	158
4. Selective production of methane and ethylene					
Cu–porphyrin complex	0.5 m KHCO_3_	−0.976 (vs RHE)	CH_4_ and C_2_H_4_ (44%)	13.2 mA cm^−2^ (CH_4_)	176
				8.4 mA cm^−2^ (C_2_H_4_)	
Cu NPs supported on glassy carbon	0.1 m NaHCO_3_	−1.25 (vs RHE)	CH_4_ (80%)	≈9 mA cm^−2^	110
Cu nanowires	0.1 m KClO_4_	−1.10 (vs RHE)	C_2_H_6_ (20.3%)	4–5 mA cm^−2^	113
	0.1 m KHCO_3_		C_2_H_6_ (17.4%)		
	0.1 m K_2_HPO_4_		C_2_H_6_ (10%)		
Plasma‐treated Cu foil	0.1 m KHCO_3_	−0.90 (vs RHE)	C_2_H_4_ (60%)	–	114
Cu foam	0.5 m NaHCO_3_	−0.80 (vs RHE)	C_2_H_4_, C_2_H_6_ (55%)	–	115
Glycine/Cu nanowires	0.1 m KHCO_3_	−1.90 (vs Ag/AgCl)	C_2_H_4_, C_2_H_6_, C_3_H_6_ (34.1%)	≈11 mA cm^−2^	116
Cu nanocubes [44 nm]	0.1 m KHCO_3_	−1.1 (vs RHE)	C_2_H_4_ (41%)	≈5.5 mA cm^−2^	119
Cu NPs	0.1 m KHCO_3_	−1.1 (vs RHE)	CH_4_ (57%)	23 mA cm^−2^	174
			C_2_H_4_ (<20%)		
			CO (<5%)		
			HCOOH (<5%)		
Pd–Au alloy	0.1 m KH_2_PO_4_/0.1 m K_2_HPO_4_	−0.60 (vs RHE)	CO (30.9%)	–	203
		−1.40 (vs RHE)	CH_4_ (2%)		
		−1.40 (vs RHE)	C_2_ hydrocarbons (0.7%)		
		−1.40 (vs RHE)	C_3_ hydrocarbons (0.3%)		
		−1.30 (vs RHE)	1‐Butene (0.16%)		
Cu_2_Pd alloy	0.1 m TBAPF_6_/CH_3_CN/1 m H_2_O	−1.8 (vs Ag/AgNO_3_)	CH_4_ (51%)	≈6 mA cm^−2^	177
Ni*_x_*Ga*_y_* alloy	0.1 m NaHCO_3_	−0.48 (vs RHE)	CH_4_ (>2%)	(−1.18 V) 140 µA cm^−2^	124
			C_2_H_4_ (1.3%)	(−1.18 V) 100 µA cm^−2^	
Cu_2_O/Cu	0.1 m KHCO_3_	−0.98 (vs RHE)	C_2_H_4_ (42.6%)	13.3 mA cm^−2^	179
			C_2_H_5_OH (11.8%)	3.7 mA cm^−2^	
			C_3_H_7_OH (5.4%)	1.7 mA cm^−2^	
Mo_2_C	0.1 m KHCO_3_	–1.10 (vs RHE)	CH_4_ (29%)	>30 mA cm^−2^	141
			H_2_ (≈39%)		
N‐doped carbon	[bmim]BF_4_/H_2_O	−1.4 (vs RHE)	CH_4_ (93.5%)	1.42 mA cm^−2^	149
Pyridinic‐N rich graphene/Cu	0.5 m KHCO_3_	−0.90 (vs RHE)	C_2_H_4_ (19%)	7.7 A g^−1^	202
5. Selective production of alcohols					
Enzymes	Phosphate buffer solution	−1.20 (vs Ag/AgCl)	CH_3_OH (≈10%)	–	180
[4‐(3‐Phenoxy‐2,2‐bis(phenoxymethyl)propoxy)pyridine]@Cu–Pd	0.5 m KCl	−0.04 (vs RHE)	CH_3_OH (26%)	21 mA cm^−2^	204
		−0.64 (vs RHE)	C_2_H_5_OH (12%)	–	
Cu nanocrystals	0.1 m KHCO_3_	−0.95 (vs RHE)	C_3_H_7_OH	1.74 mA cm^−2^	111
Mo–Bi alloy	0.5 m [bmim]BF_4_/MeCN	−0.70 (vs RHE)	CH_3_OH (71.2%)	12.1 mA cm^−2^	126
Cu_2_O	0.1 m KHCO_3_	−0.99 (vs RHE)	C_2_H_4_ (34–39%)	30–35 mA cm^−2^	178
			C_2_H_5_OH (9–16%)		
Cu_2_O	0.5 m KHCO_3_	−2.0 (vs Co_3_O_4_)	C_2_H_5_OH (96.2%)	4.5 mA cm^−2^	185
Cu_2_O/multiwalled CNT	0.5 m NaHCO_3_	−0.80 (vs RHE)	CH_3_OH (38.0%)	7.5 mA cm^−2^	188
Oxidized Cu	0.5 m KHCO_3_	−1.10 (vs SCE)	CH_3_OH (38.0%)	–	189

### 3.1 Selective Production of Formic Acid/Formates

As early as 1870, HCOOH/HCOO^−^ generations have been realized from electrocatalytic reduction of CO_2_ molecules in aqueous solution.^159^ From electrokinetic perspective, the rate‐determining step was determined to be the hydrogenation step, which realized the bonding of electrogenerated surface hydrogen onto CO_2_ molecules. The obtained *COOH intermediate was then reduced by one electron to generate the HCOO^−^ product. As aforementioned, some metal–organic complexes have been reported to mainly produce HCOOH/HCOO^−^ products with the assistance of ion liquids.^41^, ^44^ Afterward, numerous efforts have been made for the large‐scale conversion of CO_2_ to HCOOH/HCOO^−^ with economic practicability.^160^ As the first class of metals, Sn, In, Hg, Pb, and Bi based catalysts can facilitate HCOOH/HCOO^−^ generation,^101^, ^161^ due to the easy desorption of *COO^−^ intermediates on the surface. On the other hand, Cu foam with higher surface roughness can present an ultrahigh HCOOH production rate among Cu‐based electrodes, and also can suppress the formation of *CO intermediates and inhibit the evolution of dimeric products (CH_4_ and C_2_H_4_).^162^ Some metal oxides have been found to be outstanding candidates for generating HCOOH/HCOO^−^, for example, partially oxidized Co 4‐atomic‐layer^131^ could realize a HCOO^−^ selectivity of 90% owing to its good stabilization of *COO^−^ intermediates. Sn, SnO*_x_*, as well as Sn–Ag alloys also can facilitate selective *COO^−^ protonation for HCOO^−^ formation.^71^, ^72^, ^73^, ^74^, ^75^, ^163^, ^164^, ^165^ Besides, carbon nanomaterials like NCNTs, graphene, and others presented enhanced current density and high Faradaic efficiency for acid production, mainly attributed to the preferable CO_2_ adsorption and stabilization of reduced *COO^−^ intermediates.^147^, ^151^, ^155^

### 3.2 Selective Production of Carbon Monoxide

Two common issues have seriously influenced the reductive activity for CO formation: (1) the conversion of CO_2_ to *COOH is hindered by the weak *COO^−^ binding; (2) the release of CO gas from the electrocatalyst surface is suppressed by strong binding of *CO. Aiming at these problems, the effects of morphology, active sites and exposed facets of electrocatalysts (such as Au, Ag, Bi, Zn, Pd, and metal oxides) have been experimentally and computationally investigated.^63^, ^166^ Many researchers reported the synthesis of Cu–M (M = Au, In, Sn) and other metallic alloys for converting CO_2_ to CO with a low overpotential.^121^, ^122^, ^123^, ^167^ With the assistance of ionic liquid, ternary transition metal dichalcogenides (MoSeS)^168^ as well as some metal complexes like Fe/Co porphyrins,^39^, ^40^, ^169^ Mn bipyridines,^52^ and Zn phosphines,^53^ have been found to exhibit preferable catalytic activity for generating CO. Carbon materials were also utilized for selective and stable CO_2_ reduction into CO. NCNTs as a durable electrocatalyst also showed ultralow overpotential (−0.18 V) and selectivity (80%) for CO production.^144^

### 3.3 Selective Production of Formaldehyde

A binuclear cobalt complex, [Co_2_BPP], also undertook a four‐electron reduction pathway for HCHO generation below −1.0 V (vs NHE).^58^ In 1995, [M(4‐v‐tpy)_2_]^2+^ or [M(6‐v‐tpy)_2_]^2+^ (M = Cr, Ni, Co, Fe, Ru, or Os) complexes were employed to reduce CO_2_ into formaldehyde (HCHO) as the dominate product.^59^ Boron‐doped diamond, an ideal catalyst with high overpotential for hydrogen evolution, exhibited superior Faradaic efficiency of 74% for HCHO generation in methanol and aqueous solutions.^157^ Moreover, Boron‐doped diamond decorated with Cu NPs showed a high current density of ≈5.1 mA cm^−2^ at −1.3 V (vs NHE), and the highest Faradaic efficiency for HCHO evolution was expected to be 80% under optimized reaction conditions.^158^

### 3.4 Selective Production of Methane and Ethylene

For the yield of high‐energy‐density hydrocarbon products, such as CH_4_ and C_2_H_4_, reaction processes with six or more electron transfer and multiple intermediate steps at higher overpotentials are normally required. Nonprecious Cu metal as a promising electrocatalyst possesses high activities and Faradaic efficiencies for methanation/vinylation, thus can produce higher value‐added hydrocarbon products, mainly methane (CH_4_), ethylene (C_2_H_4_) in considerable amounts.^170^, ^171^, ^172^, ^173^, ^174^, ^175^, ^176^ Cu‐based alloys^177^ and Cu oxides^178^, ^179^ also have been qualified for the formation of hydrocarbons. Some other adopted electrocatalysts such as organic metal complexes, metal alloys, metal carbides, and carbon materials for selective generation of hydrocarbons are listed in Table 2.

### 3.5 Selective Production of Alcohols

The production of alcohols in CO_2_ electrocatalytic reduction suffers from low yield and poor selectivity. Many catalysts, such as enzymes, metals, metal alloys, metal oxides/chalcogenides, and CNTs have been used for generating alcohols, such as CH_3_OH and C_2_H_5_OH.

Interestingly, bio‐electrocatalytic reduction of CO_2_ promoted by immobilized enzymes realized a Faradaic efficiency of ≈10% for CH_3_OH generation.^180^ Pyridinium (PyrH^+^) cations on Pt interface were employed to generate CH_3_OH at low overpotentials.^181^ Among Pt_3_Co alloy nanostructures with different morphologies, Pt_3_Co octapods displayed the highest TOF number of 758 h^−1^ for CH_3_OH production.^182^ Mo–Bi bimetallic chalcogenide with the help of ion liquids reached a high Faradaic efficiency of 71.2% for selective CH_3_OH production.^126^ Notably, N‐doped graphene/CNTs were theoretically predicted to be a good electrocatalyst for effective CH_3_OH production at the applied potential from −1.29 to −0.49 V.^183^

There are very few existing optional electrocatalysts for the selective production of C_2_H_5_OH. Remarkably, Cu and Cu*_x_*O based catalysts have shown special selectivity for reduction of CO_2_ to C_2_/C_3_ compounds (including C_2_H_5_OH, *n*‐propanol), owing to the favorable d‐band levels.^109^, ^111^, ^113^, ^118^, ^119^, ^178^, ^184^, ^185^, ^186^, ^187^, ^188^, ^189^ Ullah et al. demonstrated that Ir/Ru oxide could efficiently convert of CO_2_ into different valuable organic molecules (ethanol as the major product; methanol, acetone and acetaldehyde as the minor products in the liquid phase).^190^ It should be noted that photo‐assisted electroreduction of CO_2_ can also selectively generate alcohols through the aid of diversified photocathodes.^191^, ^192^, ^193^, ^194^, ^195^, ^196^

## 4 Challenges and Perspectives

The above sections intensively summarized the recent considerable progress in CO_2_ electroreduction. However, to scale up the technology for practical and commercial applications, some inevitable challenges still need to be resolved. First, the kinetically sluggish multielectron transfer process during CO_2_ reduction reaction require excessive overpotential, which usually leads to relatively low energy efficiency and high power consumption.

Secondary, the product selectivity of existing electrocatalytic CO_2_ reduction systems is not satisfying so far. Although the aforementioned catalysts like metal complexes, Sn, Pb, Au, and Ag can make contributes to the generation of specific products, such as CO or HCOOH/HCOO^−^, it is still difficult to selectively produce desirable chemicals with higher commercial value, such as C_2_ or longer chain chemicals.

Third, the activity degradation is a serious problem usually originated from the instability of catalysts, especially nonnoble metal catalysts. Generally, the cathodic degradation and the inactivation of reaction sites are responsible for activity decay. As the reaction lasts for a long time, the inert intermediates or poisonous by‐products preferably deposit on catalyst surface and go against further catalysis process. Moreover, hydrogen evolution is inevitable at high applied potential because of the polarization of electrode, and thus gas bubbles form drastically on the surface of cathode. This phenomenon may decrease the effective area of electrocatalyst and accelerate the cathodic degradation.^197^

Last but not least, solid fundamental theory and optimized standard experimental systems are still absent. It is hard to precisely predict the performance of specific electrocatalysts by unsubstantial theoretical study. Besides, the reaction systems and conditions in the literatures are varied, which is detrimental to the reciprocal evaluation and comparison of different experimental cases.

In view of this, more targeted efforts should be made to improve the fundamental research of CO_2_ electroreduction. In the aspect of catalysts, it is known that many metals and compounds have been investigated, and new catalysts are burgeoning. Even so, the exploration of high‐performance catalysts is far from sufficient. For example, a whole lot of alloys and intermetallic compounds with distinctive component combinations are still waiting to be inspected. When it referred to carbon materials, the intrinsic catalytic activity, decent conductivity and high‐adjustable surface state make these materials considered as promising catalysts and support materials. What is more, it is very meaningful to design novel composite catalysts composed of different materials to play a cooperative and synergistic effect, which can definitely lead to the further enhancement of overall performance.

Moreover, to expose more desirable active sites, the morphology control, crystalline/defect engineering and surface modification of nanosized catalysts should be studied more thoroughly. Ideally, the structure of catalyst should also fulfill the requirements of high specific surface area, high carrier mobility and good long‐term durability simultaneously.

Expect for the catalyst itself, more attention should be paid on the establishment of more reliable theoretical calculation and essential electrochemical methods that can be used to understand the chemical absorption/desorption steps, the breakage/reconstruction of C=O bonds, the rate determining factor and the competition of reaction pathways. The detailed operating conditions, such as the reactor design, adopted electrolytes, separators, temperature and pressure, should be carefully compared. The experimental results combined with solid instrumental characterizations (aberration‐corrected transmission electron microscopy, electron paramagnetic resonance, scanning tunneling microscopy, synchrotron radiation X‐ray absorption spectroscopy, and so on) can come in handy to explore favorable electrocatalyst structures/components and active centers.

In brief, electrocatalytic reduction of CO_2_ into carbonic fuels and chemicals is aimed at alleviating energy and environmental problems. To overcome the critical challenges in this field, consecutive achievements like enhanced reduction activity, higher valid product efficiencies and super stability will be attained through proper research advances. Given further fundamental progress in CO_2_ reduction, the facile and clean recycling of carbon resources for renewable fuels and high‐value chemicals is expected to be realized in the future.

## Conflict of Interest

The authors declare no conflict of interest.
